# Introduction to Special Issue: Translational Research on Pain and Pain Management in Later Life

**DOI:** 10.1093/geroni/igad119

**Published:** 2023-12-12

**Authors:** M Cary Reid, Karl Pillemer

**Affiliations:** Division of Geriatrics and Palliative Medicine, Weill Cornell Medicine, New York, New York, USA; Division of Geriatrics and Palliative Medicine, Weill Cornell Medicine, New York, New York, USA; College of Human Ecology, Cornell University, Ithaca, New York, USA

The Institute of Medicine estimates that more than 100 million Americans are affected by chronic pain (Institute of Medicine (US) Committee on Advancing Pain Research, Care, and Education, 2011). Older adults are disproportionately affected, because of the high prevalence of age-associated diseases in which pain is often the primary symptom (Markis et al., 2014; Reid et al., 2015). Prevalence studies indicate that as many as 50% of older adults experience bothersome pain on a regular basis (Dahlhamer et al., 2018; Galicia-Castillo & Weiner, 2023; Larsson et al., 2017; Lin et al., 2020; Rikard et al., 2023). Causes of pain include arthritis and arthritis-related diseases, neuropathies from diabetes and shingles, as well as cancer and cancer treatments. Pain is also highly prevalent in the advanced stages of many chronic illnesses, including heart, kidney, and lung disease (Phongtankuel et al., 2016). Pain is a major reason older people seek medical care and thus a leading driver of health care costs (Cohen et al., 2021; Wagner, 2022).

Beyond its immediate effects on well-being, chronic pain is associated with multiple deleterious consequences, including reduced mobility, falls, frailty, depression and anxiety, sleep impairment, and social isolation (Domenichiello & Ramsden, 2019; Dueñas et al., 2016; Makris et al., 2014; Reid et al., 2015; Saraiva et al., 2018). The negative effects of pain extend beyond the individual, often disrupting both family and social relationships (Riffin et al., 2016; Suso-Ribera et al., 2016). Adverse effects at the societal level include decreased productivity, absenteeism, and early retirement (Fagerlund et al., 2023; McDonald et al., 2011; Saastamoinen et al., 2012). In sum, pain constitutes a threat to the health and well-being of older adults.

Although increased attention has focused on researching pain in later life, key knowledge gaps remain. An increased understanding of social, biological, and psychological factors associated with pain, current pain management practices, and ways to measure pain is needed to advance prevention and treatment approaches. Further given the continued high prevalence and burden of pain in older persons, and the movement away from primary reliance on pharmacologic treatments (Bushnell et al., 2021; Meerwijk et al., 2020; Pritchard et al., 2022), it is important to develop, test, and disseminate behavioral treatment alternatives (Driscoll et al., 2021; Niknejad et al., 2018; U.S. Department of Health and Human Services, 2019).

Behavioral approaches in the form of psychological and exercise therapies are now routinely recommended as primary treatments for chronic pain; but the evidence base regarding which approaches work best and in what types of older adults remains limited. Research is needed to develop scalable behavioral approaches that can be readily accessed in both clinical and nonclinical settings (Cohen et al., 2021; Darnall, 2021; Geneen et al., 2017; Williams et al., 2020). Further, research has documented that specific groups, that is, minority elders, cognitively impaired older adults, and those living in rural areas, remain underrepresented in pain research (Anderson et al., 2009; Brecher & West T, 2016; Eaton et al., 2018; Janevic et al., 2017; Lin et al., 2018; Prunuske et al., 2014; Rajkumar et al., 2017). Ramifications of these gaps include limited generalizability and applicability of existing study findings, lack of appropriate measures that are valid for diverse subgroups, and lack of clarity regarding whether existing interventions can meet the needs of specific subpopulations (Booker et al., 2021). Investigations that seek to understand better how race, ethnicity, geographic location, socioeconomic status, and health literacy influence the experience of pain, treatment preferences, and outcomes associated with commonly administered therapies are sorely needed. Generating new knowledge in these areas will benefit not only policy-makers and practitioners, but also older individuals affected by pain.

This special issue begins to address these knowledge gaps by shedding light on understudied factors associated with pain, new measurement approaches, treatment practices, and patterns, as well as promising behavioral approaches to mitigate pain in aging adults. It is notable that, of the 13 articles accepted for publication, 6 focus on an underrepresented population.

## Identifying Factors Associated with Pain

Andrasfay et al. (2023) analyzed Health and Retirement Study (HRS) data to examine how pain and physical job demands influence workers’ (ages 51–56) expectations regarding their ability to work beyond the ages of 62 or 65. Individuals experiencing activity-limiting pain and who worked at physically demanding jobs were much more likely to report a low expectation of working beyond age 62 or 65. Working to 70 or beyond may not be feasible for this group of older adults. These results are important in light of ongoing calls to raise the retirement age.

Yang and colleagues shed light on how incarceration, an established risk factor for poor health outcomes across the life course, affects the pain experienced in later life. Yang et al. (2023) analyzed HRS data (over the period 2012–2018) to assess associations between a history of incarceration and reporting either moderate-to-severe pain or activity-limiting pain. After controlling for relevant confounders using propensity score matching, the likelihood of reporting moderate-to-severe pain and activity-limiting pain were both significantly increased in persons with a history of incarceration. Although more research is needed to define these associations and possible causal mechanisms, the results of the study support efforts to screen for incarceration histories in health care settings.

Zhou et al. (2023) expand our understanding of the association between pain and fall worry in persons with cognitive impairment. Prior research has documented that fall worry (also referred to as fear of falling) is prevalent among persons with cognitive impairment and is a recognized risk factor for falling. Utilizing 2015 National Health and Aging Trends Study data, Zhou et al. identified 1,150 participants (mean age 81) with either mild cognitive impairment or dementia and examined associations between two pain characteristics (severity of pain and number of pain sites and their location) and nonactivity-limiting fall worry, as well as activity-limiting fall worry (defined as a worry about falling down that would limit one’s activities). The severity of pain and number of pain sites were both independently associated with a greater likelihood of endorsing activity-limiting fall worry. Both lower body and upper body pain were also independently associated with an elevated risk of activity-limiting fall worry. These results support efforts to include pain management elements in fall prevention interventions directed at older adults with cognitive impairment.

Shaw et al. (2023) explore the impact of pain in hospitalized adults with dementia, three quarters of whom were rated as having moderate-to-severe dementia. Care rejection is a common behavior that leads to poor health outcomes in this population and factors underlying this type of behavior remain poorly defined. The authors utilized validated measures to document care rejection episodes while concurrently rating both pain and delirium severity. Pain was documented far more often in patients rejecting care than delirium. Further, specific behaviors including crying, pushing away, and screaming/yelling indicated a higher level of pain severity, whereas no rejection of care behaviors was associated with delirium severity. These results highlight the importance of considering pain as a cause of care-rejecting behavior in hospitalized patients with moderate-to-severe dementia.

Finally, although diabetes mellitus is an established risk factor for multiple adverse health outcomes including painful neuropathies, relatively little research has focused on the association between pain and diabetic complications in Hispanic older adults. Onwudebe et al. (2023) analyze data from the Hispanic Established Population for the Epidemiologic Study of the Elderly to assess for associations between diabetes and two types of pain: pain on weight bearing and activity-limiting pain. Participants with at least one diabetic complication were found to have a higher likelihood of reporting pain on weight bearing and activity-limiting pain over a 6-year period when compared to participants with diabetes and no complications. These results support efforts to screen for and treat diabetes earlier as a means of decreasing complication rates in the target population as well as screening routinely for pain in those who develop diabetic complications in clinical settings. The Onwudebe et al. study is notable because of a mean study sample age of 85 years. More investigations are needed that focus on oldest-old populations given that persons ages 80 and above constitute the fastest growing demographic in the United States.

## Expanding Methods to Measure Pain

Customary pain measures require individuals to gauge the level of pain experienced over a defined period of time (e.g., past day or week) or to recall the level of pain experienced when engaging in a specific activity. These retrospective appraisals raise concerns for recall bias which could lead to either the underestimation or overestimation of pain. Wilson et al. (2023) focus on the role of measuring movement-evoked pain in a study of 92 middle-aged and older adults with established osteoarthritis of the knee who underwent total knee arthroplasty. Assessments were conducted prior to and 3 months after knee surgery. The investigators employed two established performance-based measures of physical function (6-minute walk test and the stair-climbing test) and assessed pain during the 6-minute walk test and immediately after completing the stair-climbing task. Situational catastrophizing (i.e., measurement during or directly after administration of a noxious stimulus) was also measured immediately after participants completed both tests. Decreases in movement-evoked pain and situational catastrophizing were most consistently associated with improvements in functioning as measured by the two performance-based tasks. These results highlight the value of measuring pain both during and immediately after completing performance-based tests of physical functioning along with measuring situational catastrophizing in patients with pain. The results support a growing body of literature (Corbett et al., 2019; Fullwood et al., 2021) that suggests that movement-evoked pain is a particularly important measure of musculoskeletal pain above and beyond customary retrospective pain appraisals.

## Characterizing Pain Treatment Practices and Patterns in Later Life

Defining the types and range of treatments older adults self-administer (or are prescribed) to mitigate pain can be valuable to both practitioners and policy-makers. Further, given increasing calls for the use of nonpharmacologic approaches to manage pain (Bushnell et al., 2021; Meerwijk et al., 2020; Pritchard et al., 2022), gaining a better understanding of existing research in this area would also be helpful, particularly as it relates to aging adults. Zajacova et al. (2023) analyzed online survey data collected as part of the 2020 Recovery and Resilience: COVID-19 study. The analytic sample included 2,041 U.S. and 2,072 Canadian adults ages 18 and above. Participants were asked to report on the types of treatments, medications, and self-care approaches used to treat or prevent pain. Response items included both closed-ended questions along with an open-ended question to capture additional modalities used by respondents. Use of over-the-counter medications, “just living with pain,” and exercise were the most commonly endorsed methods for managing pain, reported by 55%, 41%, and 40% of respondents, respectively. Of note, nearly 6% endorsed alcohol use as a means of treating pain. The most commonly endorsed treatment reported in response to the open-ended question was cannabis use. The investigators assessed for age differences and found that respondents ages 65+ were more likely to endorse the use of over-the-counter medications and “just living with pain” relative to younger (ages 18–44) adults. Older versus younger adults were also less likely to use complementary and alternative approaches and alcohol in an effort to treat pain. These findings expand the limited existing literature on pain management practices in the general population and reinforce the need for practitioners to inquire broadly about the methods adults use to manage pain including both adaptive and maladaptive practices.

Despite multiple guidelines discouraging the use of opioid medications as treatment for chronic noncancer pain (Dowell et al., 2022; U.S. Department of Health and Human Services, 2019; U.S. Department of Veteran Affairs, 2022), opioids continued to be widely prescribed. For example, in a 2018 Michigan National Poll on Healthy Aging survey of adults ages 50–80, 29% of respondents reported filling a prescription for an opioid medication in the past 2 years (Harbaugh et al., 2018). Fennell et al. (2023) shed new light on factors associated with opioid use in aging adults by analyzing five waves of HRS data that span a 16-year (2005–2020) period. The investigators examined a range of factors and their association with either a single wave or multiple waves of opioid use, including sociodemographic, health, and geographic variables. The analytic sample consisted of 10,713 adults ages 51 and above. Single-wave and multiwave opioid use was endorsed by 13.2% and 6.3% of participants, respectively. Relative to nonusers, both single-wave and multiwave opioid users were more likely to be younger, report lower household wealth, and live in the Midwest, South, or West, whereas multiwave users were less likely to be Black, Hispanic, or never married. These findings support efforts to ascertain possible mechanisms underlying these observed differences. Further, given the finding that the youngest age category (age 50–59) was the group at greatest risk for multiwave opioid use, targeted efforts to reduce opioid use in this age group would appear to be particularly appropriate.

Choudry et al. (2023) conducted a scoping review to characterize the types of studies that have examined nonpharmacologic pain treatments in the Medicare population with a specific focus on identifying and analyzing both qualitative as well as nonexperimental quantitative studies. The investigators conducted a comprehensive search of multiple databases over a 20-year (1992–2022) period and identified 33 studies for analysis. With respect to the type of modality examined, manipulative therapies in the form of physical therapy or chiropractic care were by far the most commonly studied treatments, whereas back pain was the most commonly studied pain type. Notable knowledge gaps identified by this scoping review include (a) a limited number of studies focused on complementary approaches such as yoga, tai chi, and multidisciplinary rehabilitation; (b) failure to report race/ethnicity data in many studies, (c) an absence of Medicare Advantage enrollees in all but one of the analyzed studies; and (d) a paucity of secular trend analyses to characterize temporal trends in the use of nonpharmacologic treatments over time. These results highlight the need for future studies that address these important knowledge gaps in studies of older adults.

## Developing Scalable Behavioral Pain Treatments for Use by Older Adults

Behavioral strategies that seek to mitigate the physical and psychological burdens of chronic pain and restore physical functioning are receiving increased attention as viable alternatives to pharmacologic approaches. Four articles in this special issue either tested a promising behavioral intervention or adapted one for use in a specific population. Ong et al. (2023) employed a pilot randomized controlled trial (RCT) design to examine the feasibility, acceptability, and preliminary efficacy of an online intervention that sought to enhance positive affect in aging adults with fibromyalgia, a population with recognized deficits in positive affect. The skill training took place over a 5-week period and included topics such as noticing positive events, employing mindfulness techniques, and use of positive reappraisal. The investigators demonstrated high retention rates and satisfaction ratings along with significant between-group differences in positive affect enhancement, as well as reductions in negative affect and pain catastrophizing at the 1-month follow-up. These findings highlight the promise of using internet-delivered interventions in populations with chronic pain to address established access barriers to psychological pain treatments.

Riffin et al. (2023) evaluated a family caregiver-directed intervention designed to enhance caregivers’ ability to assess pain among care recipients (community-dwelling older adults with moderate-to-severe dementia) and to communicate those findings to the care recipient’s physician as a way of enhancing pain care. Intervention participants received the training weekly over a 4-week period by a trained interventionist. The pilot RCT results confirmed the feasibility and acceptability of the intervention and demonstrated significant improvements in self-efficacy for communicating pain information among intervention (vs control) arm participants. These results support future efficacy testing of the intervention that holds substantial promise as a way to address the underdetection and undertreatment of pain in persons with dementia.

Taylor et al. (2023) employed a pilot RCT design to evaluate the feasibility, acceptability, and preliminary efficacy of a behavioral intervention for use by middle-aged and older African American women experiencing comorbid pain and depressive symptoms. The intervention employed behavioral activation with individual goal setting and consisted of up to eight in-person (or virtual) visits with a nurse interventionist. The investigators successfully established the intervention’s feasibility (measured by adherence to the weekly treatment sessions, retention rate, and ability of participants to set specific goals) and preliminary efficacy (measured by reduction in depressive symptom scores). These findings should be extended to testing this promising behavioral approach in a population with established inequities in pain and mental health care.

Finally, Harrison et al. (2023) report on the adaptation of an evidence-based pain communication intervention for use by older adults with cancer residing in rural areas, where undertreatment of pain is common. The investigators adapted the Cancer Health Empowerment for Living Without Pain (Ca-HELP) program, which is an evidence-based tool that enhances patients’ self-efficacy for pain communication. The adaptation involved obtaining feedback from key stakeholder groups, including patients, informal caregivers, and their health care providers. Recommendations for program modification included changes in the way the intervention is delivered, simplification of intervention materials, and the amount of content delivered in order to best meet the needs of the target population. The adapted intervention shows promise as a way to address the undertreatment of pain in older adults receiving cancer care in rural settings.

## Next Steps

The articles in this special issue point to the promise and challenge of translational research on pain. They shed light on potential short- and long-term consequences of pain, new approaches to measurement, as well as promising nonpharmacological interventions for its management. A critical need, however, is to link these areas of inquiry, moving from the production of basic knowledge about pain to evidence-based approaches to prevention and treatment. Creating a more seamless translational pipeline requires innovative approaches to stakeholder involvement, so that interventions have immediate real-world relevance. Given the continued growth in the older population, and the tremendous amount of suffering generated by untreated or undertreated pain, fostering collaboration among researchers, practitioners, and affected older people themselves should be a top priority for the field.

## References

[CIT0001] Anderson, K. O., Green, C. R., & Payne, R. (2009). Racial and ethnic disparities in pain: Causes and consequences of unequal care. The Journal of Pain, 10(12), 1187–1204. 10.1016/j.jpain.2009.10.002 19944378

[CIT0002] Andrasfay, T., Fennell, G., & Crimmins, E. (2023). Pain, physical demands at work, and future work expectations among older adults in the United States. Innovation in Aging, 7(10), igad089. 10.1093/geroni/igad089 PMC10714917 38094935

[CIT0003] Booker, S. Q., Bartley, E. J., Powell-Roach, K., Palit, S., Morais, C., Thompson, O. J., Cruz-Almeida, Y., & Fillingim, R. B. (2021). The imperative for racial equality in pain science: A way forward. The Journal of Pain, 22(12), 1578–1585. 10.1016/j.jpain.2021.06.008 34214701 PMC9133713

[CIT0004] Brecher, D. B., & West, T. L. (2016). Underrecognition and undertreatment of pain and behavioral symptoms in end-stage dementia. The American Journal of Hospice & Palliative Care, 33(3), 276–280. 10.1177/1049909114559069 25433066

[CIT0005] Bushnell, M. C., Frangos, E., & Madian, N. (2021). Non-pharmacological treatment of pain: Grand challenge and future opportunities. Frontiers in Pain Research (Lausanne, Switzerland), 2, 696783. 10.3389/fpain.2021.696783 35295445 PMC8915661

[CIT0006] Choudry, E., Rofé, K. L., Konnyu, K., Marshall, B. D. L., Shireman, T. I., Merlin, J. S., Trivedi, A. N., Schmidt, C., Bhondoekhan, F., & Moyo, P. (2023). Treatment patterns and population characteristics of nonpharmacologic management of chronic pain in the United States’ Medicare population: A scoping review. Innovation in Aging, 7(10), igad085. 10.1093/geroni/igad085 PMC10714895 38094932

[CIT0007] Cohen, S. P., Vase, L., & Hooten, W. M. (2021). Chronic pain: An update on burden, best practices, and new advances. Lancet, 397(10289), 2082–2097. 10.1016/S0140-6736(21)00393-7 34062143

[CIT0008] Corbett, D. B., Simon, C. B., Manini, T. M., George, S. Z., Riley, J. L. 3rd, & Fillingim, R. B., (2019). Movement-evoked pain: Transforming the way we understand and measure pain. Pain, 160(4), 757–761. 10.1097/j.pain.0000000000001431 30371555 PMC6424644

[CIT0009] Dahlhamer, J., Lucas, J., Zelaya, C., Nahin, R., Mackey, S., DeBar, L., Kerns, R., Von Korff, M., Porter, L., & Helmick, C. (2018). Prevalence of chronic pain and high-impact chronic pain among adults—United States, 2016. MMWR. Morbidity and Mortality Weekly Report, 67(36), 1001–1006. 10.15585/mmwr.mm6736a2 30212442 PMC6146950

[CIT0010] Darnall, B. D. (2021). Psychological treatment for chronic pain: Improving access and integration. Psychological Science in the Public Interest, 22(2), 45–51. 10.1177/15291006211033612 34541966 PMC9970761

[CIT0011] Domenichiello, A. F., & Ramsden, C. E. (2019). The silent epidemic of chronic pain in older adults. Progress in Neuro-Psychopharmacology & Biological Psychiatry, 93, 284–290. 10.1016/j.pnpbp.2019.04.006 31004724 PMC6538291

[CIT0012] Dowell, D., Ragan, K. R., Jones, C. M., Baldwin, G. T., & Chou, R. (2022). CDC clinical practice guideline for prescribing opioids for pain—United States, 2022. MMWR. Recommendations and Reports : Morbidity and Mortality Weekly Report. Recommendations and Reports, 71(3), 1–95. 10.15585/mmwr.rr7103a1 PMC9639433 36327391

[CIT0013] Driscoll, M. A., Edwards, R. R., Becker, W. C., Kaptchuk, T. J., & Kerns, R. D. (2021). Psychological interventions for the treatment of chronic pain in adults. Psychological Science in the Public Interest, 22(2), 52–95. 10.1177/15291006211008157 34541967

[CIT0014] Dueñas, M., Ojeda, B., Salazar, A., Mico, J. A., & Failde, I. (2016). A review of chronic pain impact on patients, their social environment and the health care system. Journal of Pain Research, 9, 457–467. 10.2147/JPR.S105892 27418853 PMC4935027

[CIT0015] Eaton, L. H., Langford, D. J., Meins, A. R., Rue, T., Tauben, D. J., & Doorenbos, A. Z. (2018). Use of self-management interventions for chronic pain management: A comparison between rural and nonrural residents. Pain Management Nursing 19(1), 8–13. 10.1016/j.pmn.2017.09.004 29153296 PMC5807105

[CIT0016] Fagerlund, P., Shiri, R., Suur-Uski, J., Kaartinen, S., Rahkonen, O., & Lallukka, T. (2023). Separate and joint associations of chronic pain, multisite pain and mental health with sickness absence among younger employees: A register based longitudinal study. Archives of Public Health = Archives belges de sante publique, 81(1), 97. 10.1186/s13690-023-01115-1 37248528 PMC10228037

[CIT0017] Fennell, G., Jacobson, M., & Grol-Prokopczyk, H. (2023). Predictors of multi-wave opioid use among older American adults. Innovation in Aging, 7(10), igad068. 10.1093/geroni/igad068 PMC10714904 38094934

[CIT0018] Fullwood, D., Means, S., Merriwether, E. N., Chimenti, R. L., Ahluwalia, S., & Booker, S. Q. (2021). Toward understanding Movement-Evoked Pain (MEP) and its measurement: A Scoping Review. The Clinical Journal of Pain, 37(1), 61–78. 10.1097/AJP.0000000000000891 33093342 PMC7708514

[CIT0019] Galicia-Castillo, M.C., & Weiner, D.K. (2023). *Treatment of chronic non-cancer pain in older adults*. UpToDate. Retrieved June 22, 2023 from https://www.uptodate.com/contents/treatment-of-chronic-non-cancer-pain-in-older-adults

[CIT0020] Geneen, L. J., Moore, R. A., Clarke, C., Martin, D., Colvin, L. A., & Smith, B. H. (2017). Physical activity and exercise for chronic pain in adults: An overview of Cochrane Reviews. Cochrane Database of Systematic Reviews, 1(1), CD011279. 10.1002/14651858.CD011279.pub2 28087891 PMC6469540

[CIT0021] Harbaugh, C., Malani, P., Solway, E., Singer, D., Kirch, M., Clark, S., & Waljee, J. (2018). Older adults’ experiences with opioid prescriptions. University of Michigan National Poll on Healthy Aging. http://hdl.handle.net/2027.42/145684

[CIT0022] Harrison, J., Stokes, T., Hahne, J., & Shen, M. (2023). Ca-HELP: Adaptation of a communication tool to help geriatric cancer patients in rural settings talk to their doctors about pain. Innovation in Aging, 7(10), igad087. 10.1093/geroni/igad087 PMC11025374 38638362

[CIT0023] Institute of Medicine (US) Committee on Advancing Pain Research, Care, and Education. (2011). Relieving pain in America: A blueprint for transforming prevention, care, education, and research. 10.17226/13172

[CIT0024] Janevic, M. R., McLaughlin, S. J., Heapy, A. A., Thacker, C., & Piette, J. D. (2017). Racial and socioeconomic disparities in disabling chronic pain: Findings from the health and retirement study. The Journal of Pain, 18(12), 1459–1467. 10.1016/j.jpain.2017.07.005 28760648 PMC5682226

[CIT0025] Larsson, C., Hansson, E. E., Sundquist, K., & Jakobsson, U. (2017). Chronic pain in older adults: Prevalence, incidence, and risk factors. Scandinavian Journal of Rheumatology, 46(4), 317–325. 10.1080/03009742.2016.1218543 27885914

[CIT0026] Lin, P. C., Li, C. H., Chou, P. L., Chen, Y. M., & Lin, L. C. (2018). Prevalence of pain-related diagnoses in patients with dementia: A nationwide study. Journal of Pain Research, 11, 1589–1598. 10.2147/JPR.S172875 30214270 PMC6126483

[CIT0027] Lin, T., Zhao, Y., Xia, X., Ge, N., & Yue, J. (2020). Association between frailty and chronic pain among older adults: A systematic review and meta-analysis. European Geriatric Medicine, 11(6), 945–959. 10.1007/s41999-020-00382-3 32808241

[CIT0028] Makris, U. E., Abrams, R. C., Gurland, B., & Reid, M. C. (2014). Management of persistent pain in the older patient: A clinical review. JAMA, 312(8), 825–836. 10.1001/jama.2014.9405 25157726 PMC4372897

[CIT0029] McDonald, M., DiBonaventura, M., & Ullman, S. (2011). Musculoskeletal pain in the workforce: the effects of back, arthritis, and fibromyalgia pain on quality of life and work productivity. Journal of Occupational and Environmental Medicine, 53(7), 765–770. 10.1097/JOM.0b013e318222af81 21685799

[CIT0030] Meerwijk, E. L., Larson, M. J., Schmidt, E. M., Adams, R. S., Bauer, M. R., Ritter, G. A., Buckenmaier, C., & Harris, A. H. S. (2020). Nonpharmacological treatment of army service members with chronic pain is associated with fewer adverse outcomes after transition to the Veterans Health Administration. Journal of General Internal Medicine, 35(3), 775–783. 10.1007/s11606-019-05450-4 31659663 PMC7080907

[CIT0031] Niknejad, B., Bolier, R., Henderson, C., Delgado, D., Kozlov, E., Löckenhoff, C., & Reid, M. (2018). Association between psychological interventions and chronic pain outcomes in older adults: A systematic review and meta-analysis. JAMA Internal Medicine, 178(6), 830–839. 10.1001/jamainternmed.2018.0756 29801109 PMC6145761

[CIT0032] Ong, A. D., Wilcox, K. T., Moskowitz, J. T., Wethington, E., Addington, E. L., Sanni, M. O., Kim, P., & Reid, C. (2023). Feasibility, acceptability, and preliminary efficacy of a positive affect skills intervention for adults with fibromyalgia. Innovation in Aging, 7(10), igad070. 10.1093/geroni/igad070 PMC10714916 38094931

[CIT0033] Onwudebe, C., Al Snih, S., Raji, M. A., & Milani, S. A. (2023). Diabetes complications and pain among Mexican Americans aged 80 and older. Innovation in Aging, 7(10), igad099. 10.1093/geroni/igad099 PMC10714911 38094936

[CIT0034] Phongtankuel, V., Amorapanth, P. X., & Siegler, E. L. (2016). Pain in the geriatric patient with advanced chronic disease. Clinics in Geriatric Medicine, 32(4), 651–661. 10.1016/j.cger.2016.06.008 27741961

[CIT0035] Pritchard, K. T., Baillargeon, J., Lee, W. C., Raji, M. A., & Kuo, Y. F. (2022). Trends in the use of opioids vs nonpharmacologic treatments in adults with pain, 2011-2019. JAMA Network Open, 5(11), e2240612. 10.1001/jamanetworkopen.2022.40612 36342717 PMC9641539

[CIT0036] Prunuske, J. P., St Hill, C. A., Hager, K. D., Lemieux, A. M., Swanoski, M. T., Anderson, G. W., & Lutfiyya, M. N. (2014). Opioid prescribing patterns for non-malignant chronic pain for rural versus non-rural US adults: A population-based study using 2010 NAMCS data. BMC Health Services Research, 14, 563. 10.1186/s12913-014-0563-8 25407745 PMC4241226

[CIT0037] Rajkumar, A. P., Ballard, C., Fossey, J., Orrell, M., Moniz-Cook, E., Woods, R. T., Murray, J., Whitaker, R., Stafford, J., Knapp, M., Romeo, R., Woodward-Carlton, B., Khan, Z., Testad, I., & Corbett, A. (2017). Epidemiology of pain in people with dementia living in care homes: Longitudinal course, prevalence, and treatment implications. Journal of the American Medical Directors Association, 18(5), 453.e1–453.e6. 10.1016/j.jamda.2017.01.024 28330634

[CIT0038] Reid, M. C., Eccleston, C., & Pillemer, K. (2015). Management of chronic pain in older adults. BMJ, 350, h532. 10.1136/bmj.h532 25680884 PMC4707527

[CIT0039] Riffin, C., Brody, L., Mukhi, P., Herr, K., Pillemer, K., Rogers, M., Henderson, C. R., & Reid, C. (2023). Establishing the feasibility and acceptability of a caregiver targeted intervention to improve pain assessment among persons with dementia. Innovation in Aging, 7(10), igad074. 10.1093/geroni/igad074 PMC10714902 38094933

[CIT0040] Riffin, C., Fried, T., & Pillemer, K. (2016). Impact of pain on family members and caregivers of geriatric patients. Clinics in Geriatric Medicine, 32(4), 663–675. 10.1016/j.cger.2016.06.010 27741962 PMC5116301

[CIT0041] Rikard, S. M., Strahan, A. E., Schmit, K. M., & Guy, G. P. (2023). Chronic pain among adults—United States, 2019-2021. MMWR Morbidity and Mortality Weekly Report, 72(15), 379–385. 10.15585/mmwr.mm7215a1 37053114 PMC10121254

[CIT0042] Saastamoinen, P., Laaksonen, M., Kääriä, S. M., Lallukka, T., Leino-Arjas, P., Rahkonen, O., & Lahelma, E. (2012). Pain and disability retirement: A prospective cohort study. Pain, 153(3), 526–531. 10.1016/j.pain.2011.11.005 22340946

[CIT0043] Saraiva, M. D., Suzuki, G. S., Lin, S. M., de Andrade, D. C., Jacob-Filho, W., & Suemoto, C. K. (2018). Persistent pain is a risk factor for frailty: A systematic review and meta-analysis from prospective longitudinal studies. Age & Ageing, 47(6), 785–793. 10.1093/ageing/afy104 30052700

[CIT0044] Shaw, C., Ward, C., Williams, A., Lee, K., & Herr, K. (2023). The relationship between rejection of care behaviors and pain and delirium severity in hospital dementia care. Innovation in Aging, 7(10), igad076. 10.1093/geroni/igad076 PMC10714906 38094937

[CIT0045] Suso-Ribera, C., Yakobov, E., & Ribera-Canudas, M. V. (2016). Coping is important for spouses too: Impulsive coping moderates the relationship between spouses’ perception of the patients’ pain intensity and spouses’ physical health. The Clinical Journal of Pain, 32(9), 755–762. 10.1097/AJP.0000000000000328 26710226

[CIT0046] Taylor, J., Clair, C. A., Gitlin, L. N., Atkins, S., Bandeen-Roche, K., Abshire Saylor, M., Hladek, M. D., Riser, T. J., Thorpe, R. J., & Szanton, S. L. (2023). Acceptability and feasibility of a pain and depressive symptoms management intervention in middle-aged and older African American women. Innovation in Aging, 7(10), igad096. 10.1093/geroni/igad096 PMC10714909 38094930

[CIT0047] U.S. Department of Health and Human Services (2019, May). Pain Management Best Practices Inter-Agency Task Force Report: updates, gaps, inconsistencies, and recommendations. https://www.hhs.gov/opioids/prevention/pain-management-options/index.html 10.3109/15360288.2015.1037530 26095483

[CIT0048] U.S. Department of Veterans Affairs (2022). Use of opioids in the management of chronic pain. https://www.healthquality.va.gov/guidelines/pain/cot/

[CIT0049] Wager, T. D. (2022). Managing pain. Cerebrum, 2022, cer-03-22. PMC9224345 35813306

[CIT0050] Williams, A. C. C., Fisher, E., Hearn, L., & Eccleston, C. (2020). Psychological therapies for the management of chronic pain (excluding headache) in adults. The Cochrane Database of Systematic Reviews, 8(8), CD007407. 10.1002/14651858.CD007407.pub4 32794606 PMC7437545

[CIT0051] Wilson, J., Madden, V. J., Pester, B. D., Yoon, J., Papianou, L. N., Meints, S. M., Campbell, C. M., Smith, M. T., Haythornthwaite, J. A., Edwards, R. R., & Schreiber, K. L. (2023). Change in pain during physical activity following total knee arthroplasty: Associations with improved physical function and decreased situational pain catastrophizing. Innovation in Aging, 7(10), igad045. 10.1093/geroni/igad045 PMC10714905 38094929

[CIT0052] Yang, Y., Lutz, G., Zhang, Y., Chen, C., & Kheirbek, R. E. (2023). The hidden toll of incarceration: Exploring the link between incarceration histories and pain outcomes among older adults in the United States. Innovation in Aging, 7(10), igad116. 10.1093/geroni/igad116 PMC10714910 38094938

[CIT0053] Zajacova, A., Pereira Filho, A., Limani, M., Grol-Prokopczyk, H., Scherbakov, D., Fillingim, R. B., Hayward, M. D., Gilron, I., & Macfarlane, G. J. (2023). Self-reported pain treatment practices among US and Canadian adults: Findings from a population survey. Innovation in Aging, 7(10), igad103. 10.1093/geroni/igad103 PMC10714903 38094928

[CIT0054] Zhou, Y., Choi, N. G., Sadak, T., Ghosh, N., & Phelan, E. A. (2023). Association between pain and fall worry among community-dwelling older people with cognitive impairment in the United States. Innovation in Aging, 7(10), igad100. 10.1093/geroni/igad100 PMC10714914 38094927

